# A new classification of TKA periprosthetic femur fractures considering the implant type

**DOI:** 10.1186/s12891-017-1855-z

**Published:** 2017-11-25

**Authors:** Johannes K. M. Fakler, Cathleen Pönick, Melanie Edel, Robert Möbius, Alexander Giselher Brand, Andreas Roth, Christoph Josten, Dirk Zajonz

**Affiliations:** 1 Department of Orthopaedic Surgery, Traumatology and Plastic Surgery, University Hospital Leipzig, Liebigstrasse 20, D-04103 Leipzig, Germany; 2 ZESBO – Center for Research on Musculoskeletal Systems, University of Leipzig, Semmelweisstrasse 14, D-04103 Leipzig, Germany

**Keywords:** Implant-dependent classification, Periprosthetic fractures, Distal femur, Total knee arthroplasty

## Abstract

**Background:**

The treatment aims of periprosthetic fractures (PPF) of the distal femur are a gentle stabilization, an early load-bearing capacity and a rapid postoperative mobilization of the affected patients. For the therapy planning of PPF a standardized classification is necessary which leads to a clear and safe therapy recommendation. Despite different established classifications, there is none that includes the types of prosthesis used in the assessment. For this purpose, the objective of this work is to create a new more extensive fracture and implant-related classification of periprosthetic fractures of the distal femur based on available classifications which allows distinct therapeutic recommendations.

**Methods:**

In a retrospective analysis all patients who were treated in the University Hospital Leipzig from 2010 to 2016 due to a distal femur fracture with total knee arthroplasty (TKA) were established. To create an implant-associated classification the cases were discussed in a panel of experienced orthopaedists and well-practiced traumatologists with a great knowledge in the field of endoprosthetics and fracture care. In this context, two experienced surgeons classified 55 consecutive fractures according to Su et al., Lewis and Rorabeck and by the new created classification. In this regard, the interobserver reliability was determined for two independent raters in terms of Cohen Kappa.

**Results:**

On the basis of the most widely recognized classifications of Su et al. as well as Lewis and Rorabeck, we established an implant-dependent classification for PPF of the distal femur. In accordance with the two stated classifications four fracture types were created and defined. Moreover, the four most frequent prosthesis types were integrated. Finally, a new classification with 16 subtypes was generated based on four types of fracture and four types of prosthesis. Considering all cases the presented implant-associated classification (κ = 0.74) showed a considerably higher interobserver reliability compared to the other classifications of Su et al. (κ = 0.39) as well as Lewis and Rorabeck (κ = 0.31). Excluding the cases which were only assessable by the new classification, it still shows a higher interobserver reliability (κ = 0.70) than the other ones (κ = 0.63 or κ = 0.45).

**Conclusions:**

The new classification system for PPF of the distal femur following TKA considers fracture location and implant type. It is easy to use, shows agood interobserver reliability and allows conclusions to be drawn on treatment recommendations. Moreover, further studies on the evaluation of the classification are necessary and planned.

## Background

The increased number of performed TKAs combined with longer implant survival and consecutive biomechanical changes of the adjacent bone is associated with a growing number of late complications [1]. The number of revisions after total knee arthroplasty increased by 40%, whereas total hip arthroplasty (THA) revisions increased by only 23% between 2006 and 2010 in the Nationwide Inpatient Sample (NIS) of the USA. During the same period both the primary TKA and the primary THA increased by only 31% [2, 3]. The most frequent reason for revisions in knee endoprosthetics is aseptic loosening due to osteolysis [2, 4]. Polyethylene abrasion is discussed as a main cause of osteolysis with a resulting implant loosening [5, 6]. Besides endoprosthetic loosening, reduced bone quality through osteoporosis and physical impairment of geriatric patients with a tendency to fall are related to an increased periprosthetic fracture risk [7, 8]. The incidence of PPF in knee prostheses varies between 0.6% and 5.5% in current literature [9–11]. For most of geriatric pre-disabled people PPF have serious consequences. With the duration of immobilization the risk for nosocomial infections, thromboses and embolisms rises and thus the mortality increases [12, 13]. In this context, conservative therapy does not serve as clinical standard due to its low efficiency which is based on often longer persisting or not guaranteed bony healing [14]. In a large meta-analysis the failure rates were partially indicated as very high with up to 50%. Especially the non-union rates vary between 1.5% and 29%, whereas the corresponding revision rates range from 4.6 to 40% [15]. Treatment aims are a gentle stabilization, an early load-bearing capacity and a rapid postoperative mobilization of the affected patients. In addition to a well-founded understanding of the possibilities of treating these fractures, which require a case-adapted and individualized therapy in principal, a clear procedure is needed. Therefore, a standardized classification of PPF, that allows distinct and safe therapy recommendations, is helpful for creating such a specific therapy concept. The first classification of supracondylar femoral fractures in knee endoprostheses dates from 1967 by Neer and colleagues [16]. It only considers the dislocation and is rarely used today. In 1991, DiGioia and Rubash extended this classification for bone quality, fracture orientation and extent of dislocation [16, 17]. Nevertheless, just like Neer it is only used in rare cases. In contrast, a widely accepted classification with therapeutic recommendations was presented by Lewis and Rorabeck in 1997. Here, the degree of dislocation and the stability of the prosthesis play a decisive role [18]. Currently, one of the most common used and newest classifications of PPF of the knee is described by Su et al. in 2003. The three types of this classification are based on a simple anatomical assignment: **type I:** fractures are proximal to the femoral component, **type II:** fracture starts at the proximal border of the femoral component and extends proximally, **type III:** all fracture parts are below the proximal border of the anterior prosthesis shield [19]. However, none of these classifications includes the various types of endoprosthesis in their assessment. In recent years, the importance of endoprosthesis revision has steadily increased and complex revision systems are widespread in the current patient population. The differently constructed systems with individual specifications have a significant influence on the surgical supply of the occurred PPF [2, 3]. In particular, technical developments for the medical treatment of periprosthetic fractures in revision systems have become established. The successful clinical usage of different investigations such as retrograde intramedullary nails with angular stable locking options, plate osteosynthesis with multidirectional locking options or polyaxial locking compression plates (LCP) as well as attachment plates has also often been described in the literature [20–24]. Based on the increase of different types of prosthesis with an accumulation of revision systems and the development of new care strategies it is necessary to create a novel more extensive classification with a clear reference to the implant.

The intention of this work is to generate a new fracture and implant-related classification of periprosthetic TKA fractures of the femur on the basis of available classifications and through a retrospective analysis of specific patients from the University Hospital Leipzig. Moreover, it is intended to accomplish a classification from which clear therapeutic recommendations can be derived.

## Methods

Prior to the start of the investigation, the local university’s ethics committee was consulted and after examination a positive vote was issued. The vote-number of the audit authority is 044/14032016. The written, informed consent was obtained from all study participants, including their consent for publication of the results. By means of a retrospective analysis all patients who were treated due to a femur fracture in the University Hospital Leipzig from 2010 to 2016 were identified (1468 patients). Subsequently, all people with a periprosthetic fracture of the femur were determined (178 patients). Excluding all femur fractures with THA, 55 TKAs with PPF of the distal femur could be recorded.

All available data were obtained from the patient documentation system which contains archived records and electronic files in IS-H SAP (Siemens AG Healthcare Sector, Erlangen, Germany), radiological findings as well as images from SIENET MagicWeb/ACOM (Siemens AG Healthcare Sector, Erlangen, Germany), among other things. Based on this, we examined each fracture localization and configuration. Furthermore, the descriptions of the fractures which were noted in the operational report were analyzed. In this context, fracture size, bone quality, stability of the prosthesis and type of care were documented. The cases were discussed in a panel of four senior orthopaedic surgeons experienced in adult reconstruction and orthopaedic trauma surgery and with a great knowledge in the field of endoprosthetics as well as fracture care. Within the scope of this case discussion, a classification together with therapeutic recommendations were derived and formulated.

### Implant-dependent classification of periprosthetic fractures of the distal femur

Based on the most widely recognized classifications of Su et al. as well as Lewis and Rorabeck, we have established an implant-dependent classification for periprosthetic fractures of the distal femur relating to the most common types of prosthesis [18, 19]. In this context four fracture types (I – IV) were created and defined relevant to the already mentioned classifications.

- Type I: Fracture is distant from the TKA (proximal of the femoral component) referring to Su type I
- Type II: Fracture starts at the level of the proximal border of TKA and extends proximally referring to Su type II
- Type III: All fracture parts are below the proximal prosthesis border referring to Su type III
- Type IV: Supracondylar fracture with prosthetic loosening referring to Lewis and Rorabeck type III

In addition, the most frequent prosthesis types (A - D) were taken into account and summarized in four groups which are defined as follows:

- Type A: bicondylar uncoupled endoprosthesis (surface replacement)
- Type B: Semi-constraint bicondylar sledges prosthesis (posterior stabilized)
- Type C: Constraint prosthesis with intramedullary anchoring
- Type D: Distal femur replacement

**Fig. 1:**
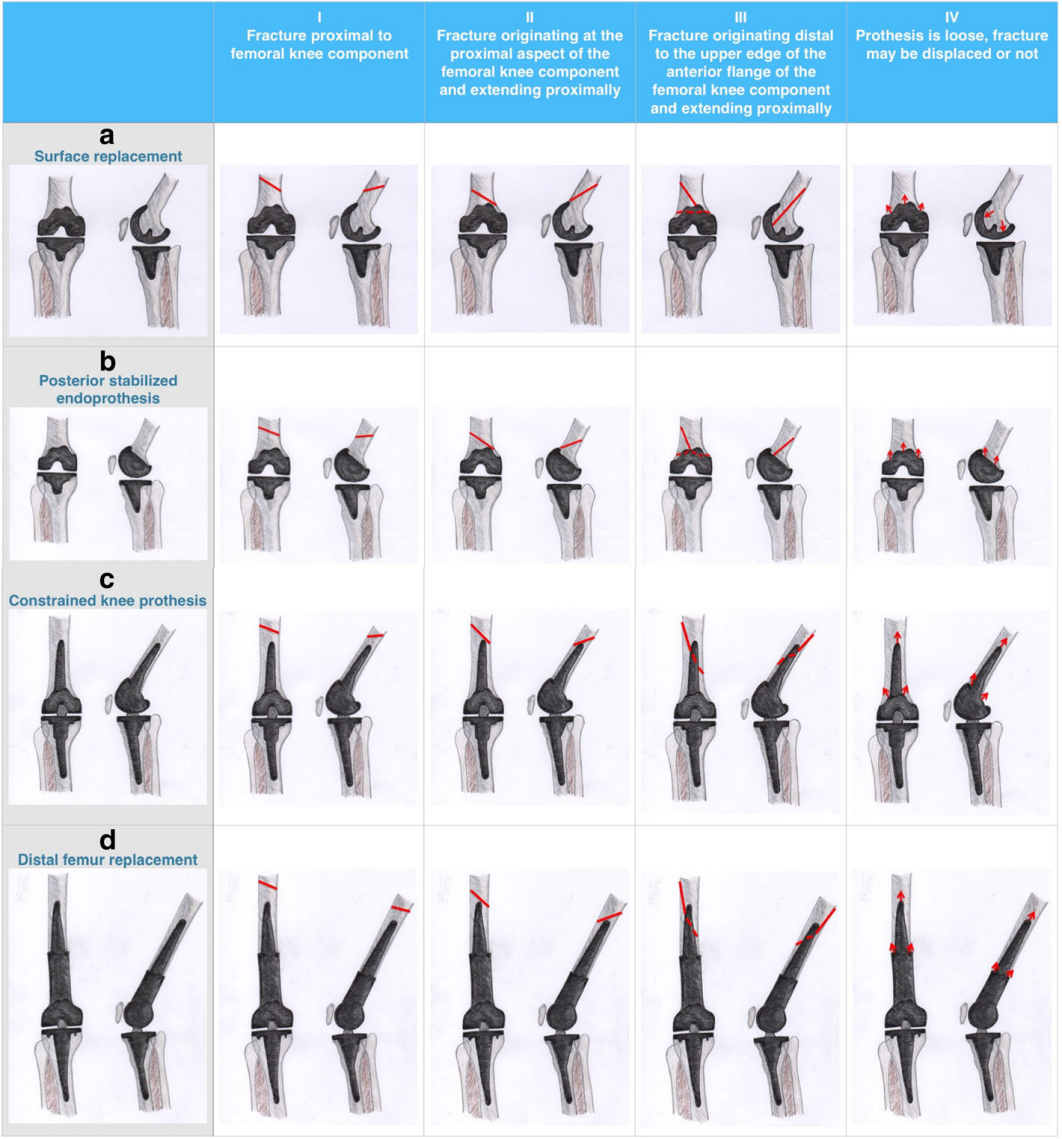
Schematic representation of the implant-dependent classification for periprosthetic fractures of the distal femur **a**: Unconstrained bikondylär TKA, **b**: posterior stabilized TKA, **c**: constrained (rotating-hinge) TKA, **d**: Distal femoral replacement. I-III: Location and expansion of fracture, IV: fracture with implant loosening. Red line depicts fracture line

Finally, a classification with 16 subtypes was made based on four types of fracture and four groups of prostheses. A schematic representation of the new implant-dependent classification of periprosthetic fractures of the distal femur is shown in Fig. 1.

### Classification-related therapeutic recommendations

For the purpose of creating classification-related therapeutic recommendations all relevant cases were assessed with regard to the new classification and appropriate treatment recommendations were derived. These recommendations are based on the expertise of the experts and the analysis of current literature. The recommendations were formulated in first, second, and third line therapy. A corresponding overview is given in Table 1.

**Table 1:** Treatment recommendations in gradation with respect to the classification of the fracture

	I	II	III	IV
A	Locking plate, retrograde nail, (antegrade nail)	Locking plate, retrograde nail,	Locking plate, revision arthroplasty (constraint endoprosthesis, eventually distal femoral replacement)	Revision arthroplasty (constraint endoprosthesis, eventually distal femoral replacement)
B	Locking plate, antegrade nail	Locking plate, revision arthroplasty (distal femoral replacement, eventually constraint endoprosthesis)	revision arthroplasty (distal femoral replacement)	revision arthroplasty (distal femoral replacement)
C	Locking plate (polyaxial, attachment plates), revision arthroplasty (distal femoral replacement)	Locking plate (polyaxial, attachment plates), revision arthroplasty (distal femoral replacement)	revision arthroplasty (distal femoral replacement)	revision arthroplasty (distal femoral replacement)
D	Locking plate (polyaxial, attachment plates), revision arthroplasty (distal femoral replacement)	revision arthroplasty (distal femoral replacement), Locking plate (polyaxial, attachment plates),	revision arthroplasty (distal femoral replacement)	revision arthroplasty (distal femoral replacement)

Finally, two senior orthopaedic surgeons classified independently the conventional X-ray images of the chosen fractures (*n* = 55) according to Su et al., Lewis and Rorabeck plus the new classification [18, 19]. With regard to the different classifications, the interobserver reliability was determined for two independent raters pursuant to Cohen Kappa [25]. On this occasion, Kappa was defined by Landis and Koch as follows: κ < 0 poor agreement, κ = 0–0.20 slight, κ = 0.21–0.40 fair, κ = 0.41–0.60 moderate, κ = 0.61–0.80 substantial, κ = 0.81–1.00 (almost) perfect [26].

## Results

The interobserver reliability for two independent raters of the three classifications (Su et al., Rorabeck et al. and implant-associated classification) is presented in Table 2. Since implant group C and D are not clearly defined by the classifications of Su et al. as well as Lewis and Rorabeck, they are excluded from the investigation. Therefore, only 41 cases were taken into account, whereas the implant-related classification could be applied in all 55 cases (κ = 0.74). As a result, the new classification showed the highest interobserver reliability (κ = 0.70). While Su et al. got similar results (κ = 0.63), just a moderate agreement could be verified by Lewis and Rorabeck (κ = 0.45).

**Table 2:** Interobserver reliability of all classifications (Su et al., Lewis and Rorabeck, new implant-related classification) according to Cohen Kappa for two independent testers with: κ < 0 poor agreement, κ = 0–0.20 slight, κ = 0.21–0.40 fair, κ = 0.41–0.60 moderate, κ = 0.61–0.80 substantial, κ = 0.81–1.00 (almost) perfect

interobserver reliability (Kappa)	Su et al. classification	Lewis & Rorabeck classification	new implant-associated classification
without exclusion (*n* = 55)	0.388 (fair)	0.309 (fair)	0.743 (substantial)
with exclusion (*n* = 41)	0.633 (substantial)	0.445 (moderate)	0.696 (substantial)

Exclusion is based on implant typ C and D, which are only defined by the new classification

## Discussion

Periprosthetic fractures in patient’s TKA show an incidence of 0.3–5.5% and after revision surgery even up to 6% [9, 10]. However, data of most studies are of reduced significance due to their small number of cases. By means of a meta-analysis of Probst and colleagues, including 55 studies with 1370 patients in total, an average number of 25 patients was obtained [27]. Despite the rarity, the incidence increases with the raise in primary knee and relevant revision endoprosthetics [2, 3]. Moreover, these are serious diagnoses, which should be treated in centers with experienced orthopaedists and traumatologists. In general, the treatment of periprosthetic fractures requires an optimal combination of endoprosthetics and fracture care [9, 12, 17]. The aim in this is to restore the limb’s original functional capability for achieving a rapid recovery of mobility and to avoid complications like bedrest of the often multimorbid patients. For this purpose and to ensure a safe treatment, a clear acute care concept with an individually adapted treatment is indispensable. For the treatment of distal thigh fractures good and clear guidelines already exist [28–30]. In this context, the classifiable treatment is established and well evaluated, too [24, 31, 32]. There are also many different classifications for the treatment of periprosthetic distal femoral fractures with clear therapeutic deductions [16, 18, 19, 33, 34]. A meta-analysis of Ebraheim and colleagues, containing 41 articles with 448 fractures, revealed Rorabeck type II as the most common fracture. Standard treatments for these types of fracture are locked plating and intramedullary nailing with similar healing rates of 87% and 84%, respectively [35]. However, despite intensive literature research, we were unable to find a classification which also refers to the type of prosthesis in addition to the fracture configuration. Although some classifications involve the loosening of the femoral component, no clear statements are made about the type of prosthesis [18]. Moreover, the range of prosthesis types has expanded considerably due to the increasing number of endoprosthesis replacements. Thus, it appears that an establishment of a classification, which relates in particular to the prosthesis properties, is necessary. For this purpose, the presented implant-dependent classification for periprosthetic fractures of the distal femur involves both the fracture localization and orientation according to Su et al. as well as the loosening of the implant relating to Lewis and Rorabeck [18, 19]. Based on this, these specifications combined with the four prosthesis types are grouped into 16 subtypes (Fig. 1). Due to the independent evaluation of two experienced orthopaedic surgeons, we were able to prove that the presented implant-associated classification shows a higher interobserver reliability compared to the classifications of Su et al. or Lewis and Rorabeck, respectively (Table 2). A major advantage of the new classification is the inclusion of the implant types C and D, which are not defined by the other ones [18, 19]. The classification of loosening is generally problematic, because of their limited detectability using conventional X-ray imaging. Most inconsistencies of our investigators were based on this fact. For this purpose, the intraoperative findings are always essential.

**Fig. 2:**
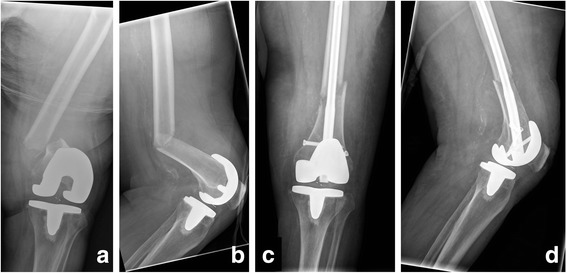
X-ray images of a 92-year-old woman with a periprosthetic fracture of the left femur after cruciate retaining (CR) bicondylar TKA (type A I); **a**: anterior-posterior and **b**: lateral radiation path with representation of the fracture before supply; **c**: anterior-posterior and **d**: lateral radiation path after reconstruction and supply by retrograde nail osteosynthesis

**Fig. 3:**
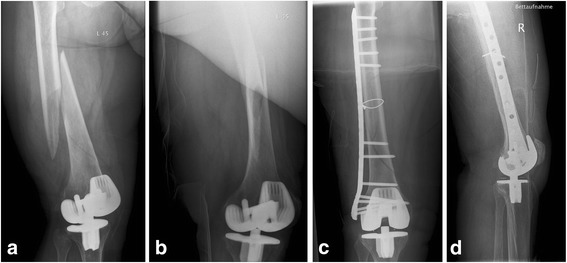
X-ray images of an 84-year-old woman with a periprosthetic fracture of the right femur with posterior stabilized (PS) TKA (type B I); **a**: anterior-posterior and **b**: lateral radiation path with representation of the fracture before supply; **c**: anterior-posterior and **d**: lateral radiation path after reconstruction and supply by plate osteosynthesis. The insertions of the distal screws were complicated by the box

**Fig. 4:**
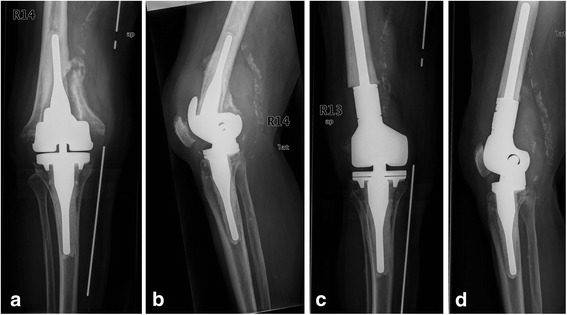
X-ray images of a 77-year-old woman with a periprosthetic fracture of the right femur with loose constrained TKA and intramedullary stem (type C IV); **a**: anterior-posterior and **b**: lateral radiation path with representation of the fracture before supply; **c**: anterior-posterior and **d**: lateral radiation path after implantation of a modular TKA

**Fig. 5:**
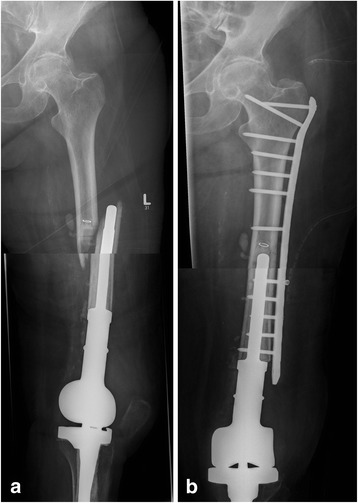
X-ray images of an 82-year-old woman with a periprosthetic fracture of the/left femur with distal femoral replacement; (Type D II) **a**: anterior-posterior radiation path with representation of the fracture before supply; **b**: anterior-posterior radiation path after reconstruction and supply by plate osteosynthesis

As already shown, therapy is a patient-specific process which presupposes a high level of expertise and experience. Therefore, a dogmatic therapy recommendation is not always effective. Type A I fracture, for example, shows that an absolute recommendation for intramedullary nails is only possible to a limited extent. On the one hand, different types of prosthesis, varying in depth of their intercondylar boxes, exist. Thus, prostheses with a widely dorsally drawn box complicate the choice of the correct nail’s point of intersection. On the other hand, there are prosthetic designs with a smaller intercondylar distance of the femoral component than the nail diameter [36]. In these cases, retrograde intramedullary nailing of periprosthetic fractures of the distal femur is not or only partly feasible. Furthermore, more than 40° deficit in knee flexion may be an obstacle for the insertion of a nail [37]. Additionally, a cemented anchoring of the femoral component can make the insertion or locking of the nail more difficult or impossible. Here, an angle stable plate osteosynthesis is more useful (Fig. 2). In type B fractures (posterior stabilized TKA) a retrograde nail osteosynthesis is impossible in most systems because of the closed design of the box. However, there also exist PS-systems in which retrograde nailing is feasible due to the open box (NexGen or Persona by Zimmer). Nevertheless, on the basis of the box the entry point can also lead to difficulties in these systems. Special nails for osteosynthesis with TKAs can simplify this problem. Especially in typ C II and III fractures, which extend far distally, osteosynthesis with plates is complex. In this context, the insertion of the distal screws is hampered by the box’s configuration in particular (Fig. 3). In these cases and in case of loosening (type IV fractures), only an implant revision often remains as ultima ratio (Fig. 4). If modular endoprostheses fail a revision is frequently indicated as well. Here, the only available treatment is a larger modular mega-endoprosthesis. In order to preserve remaining bone stock an osteosynthesis should be aspired (Fig. 5). These few examples already show the complexity of PPF’s operative care, which can also be only partially covered by the new classification. There still remains a single case decision which depends on additional multiple factors, such as bone structure (osteoporosis), cementation, stature of the patient, surgeon’s experience, etc. [38, 39]. The presented classification is just intended to ease the decision-making process, which must be adapted to each individual case.

A further aim of our working group is to evaluate the classification by means of retrospective investigations followed by prospective ones.

## Conclusions

We present an implant-dependent classification for periprosthetic fractures of the distal femur. Based on the widespread and established classifications according to Su et al. as well as Lewis and Rorabeck we created a more detailed one. In favour of a better categorization the four classic prosthesis types are additionally included. Thus, fractures can not only be classified by their anatomical assignment but in combination with the existing prosthesis. Therefore, clear treatment recommendations can be easier derived from the presented 16 subtypes, taking the individual situation into account. Further studies on the evaluation of the classification are necessary and planned.

## Abbreviations

LCP: Locking compression plates
NIS: Nationwide Inpatient Sample
PPF: Periprosthetic fractures
THA: Total hip arthroplasty
TKA: Total knee arthroplasty
